# Molecular characterization of a ceftriaxone-resistant *Neisseria gonorrhoeae* strain found in Switzerland: a case report

**DOI:** 10.1186/s12941-021-00456-5

**Published:** 2021-08-06

**Authors:** Konrad Egli, Anna Roditscheff, Ursula Flückiger, Martin Risch, Lorenz Risch, Thomas Bodmer

**Affiliations:** 1 Centre of Laboratory Medicine (CLM) Dr Risch, 3097 Liebefeld, Switzerland; 2 Zentrum Für Innere Medizin, Hirslanden Klinik Aarau, 5000 Aarau, Switzerland; 3 Centre of Laboratory Medicine (CLM) Dr Risch, 9470 Buchs, Switzerland; 4 Private University of the Principality of Liechtenstein, 9495 Triesen, Liechtenstein

**Keywords:** *Neisseria gonorrhoeae*, Phenotypic resistance, Mutations of target genes, Ceftriaxone resistance

## Abstract

**Background:**

The resistance of *Neisseria gonorrhoeae* to ceftriaxone is unusual in Switzerland. The underlying genotype responsible for resistance is suspected to be novel. Generally, resistance in *Neisseria gonorrhoeae* (Ng) involves a comprehensive set of genes with many different mutations leading to resistance to different β-lactams and fluoroquinolones.

**Case presentation:**

A patient had a positive result from specific PCR for Ng. We routinely culture all clinical specimens with a positive NG-PCR. In this particular case, we isolated a strain with resistance to ceftriaxone in Switzerland. A total of seven different genes (*penA*, *ponA*, *porinB*, *mtr*, *gyrA*, *parC*, 23S rRNA gene) in this strain were partially sequenced for comparison with phenotypic susceptibility testing. Interestingly, two different mutations in the *porinB* gene were observed, and data on this gene are limited. Information on the identified allele type of the *penA* gene is very limited as well. Three different mutations of *parC* and *gyrA* that correlate with ciprofloxacin resistance were found. The combination of ceftriaxone and ciprofloxacin resistance makes an appropriate treatment difficult to obtain due to multidrug resistance.

**Conclusion:**

The combined results for all genes show the appearance of new mutations in central Europe either due to worldwide spread or the emergence of new genetic combinations of mutations.

## Background

The increased antibiotic resistance of *Neisseria gonorrhoeae* (Ng) isolates hinders the treatment of Ng infections. Decreased susceptibility of Ng to cefixime and ceftriaxone has been reported in Switzerland; however, very few clinical specimens have a MIC ≥ 0.25 mg/l [1], which is above the EUCAST (European Committee on Antimicrobial Susceptibility Testing) breakpoint of 0.125 mg/l [2]. Ceftriaxone resistance in other European countries is also rare [3], 4. There are few reports on the molecular characterization of different resistance genes/mechanisms of Ng [3, 5–9] from different continents. Here, we describe the molecular characterization of an Ng strain found in Switzerland with an unusual combination of mutations in relevant genes. Current recommendations for treatment suggest dual therapy with azithromycin and ceftriaxone or ceftriaxone monotherapy for uncomplicated Ng infection when antimicrobial susceptibility is unknown [10]. Therefore, a ceftriaxone-resistant strain, as described here, restricts these established recommendations for first-line treatment.

## Case presentation

In December 2019, the patient presented himself to an emergency room of a Swiss hospital with a painful glans penis, showing signs of local inflammation, urethral discharge and inguinal lymphadenopathy. One week prior to symptom development, he had protected intercourse and unprotected oral sex with a female partner, of whom the health status was unknown. Furthermore, it is not known whether he had a history of sexually transmitted infections or if he received prior treatment with antibiotics. Based on his clinical symptoms, *Chlamydia trachomatis* (Ct)-, human immunodeficiency virus (HIV)-, *Treponema pallidum* (Tp)-, and Ng infection were included in the differential diagnosis. Due to a suspected Ct infection, empirical antibiotic treatment was started with 100 mg oral doxycycline two times a day for seven days. Prior to treatment, a urethral swab was sampled to check for Ct and Ng using the multiplex real-time Cobas^®^ CT/NG assay on the Cobas 6800 system (Roche Molecular Diagnostics, Pleasanton, CA, USA). We do not know whether the antibiotic therapy was adjusted according to current treatment guidelines for Ng infections or whether a test of cure (TOC) was performed since the patient is currently lost to follow-up.

Screening for Ng was performed by PCR. Clinical specimens with a positive PCR were subsequently cultured using Chocolate agar PolyViteX VCAT3 and Columbia agar + 5% sheep blood (both Biomerieux, Switzerland). In this case, a urethral swab was cultured and tested against different antibiotics (see Table 1). Susceptibility testing was performed according EUCAST 2019 [2]. Due to resistance to ceftriaxone and ciprofloxacin, different relevant genes were sequenced (see Table 2).

**Table 1:** Reported susceptibility data (MIC) of culture (according to EUCAST 2019) as well as corresponding classes of antibiotics and/or target genes

Substance	MIC	Reported phenotype	Antimicrobial/target gene^a^	Remarks
Ceftriaxone	0.25 mg/l	resistant	β-Lactam *penA*, *porinB*, *ponA*, *bla*	0.38: value from CLM Dr Risch Group 0.25: value from IMM (UZH) MIC breakpoint: S ≤ 0.125 mg/l, R > 0.125 mg/l
Penicillin	0.5 mg/l	intermediate	*PorinB*	
Ciprofloxacin	12.0 mg/l	resistant	Fluoroquinolone *gyrA* and *parC*	MIC breakpoint: S ≤ 0.03 mg/l, R > 0.06 mg/l
Azithromycin	0.5 mg/l		Macrolide *23S r*R*NA* gene	Azithromycin is always used in conjunction with another effective agent. ECOFF: 1 mg/l
Tetracycline	0.38 mg/l	susceptible	Tetracycline *rpsJ*, *mtrR*, *penB*, *tetM*-encoding plasmids	MIC breakpoint: S ≤ 0.5 mg/l, R > 1.0 mg/l

Ceftriaxone testing was performed at the CLM Dr Risch Group as well as at the Institute of Medical Microbiology (IMM) of the University of Zurich (UZH) for confirmation. ECOFF is the “epidemiological cutoff value”

^a^More details on target genes are provided in reviews of the literature [11, 12].

**Table 2:** Overview of observed sequences based on NG-STAR and PubMLST

Locus	Contig	Allele-type	Length (bp)	Start position	End position	Comments
penA^a^	*penA*	148	607	NA	NA	*penA* type 148 NonMosaic; A517G
NG_ponA	*ponA*	1	75	218	292	Mutation: L421P
NG_porB	*porinB*	55	30	116	145	Mutations: G120N, A121G
^pro^NEIS1635	*mtr*	3	66	146	211	Adenine deletion in promoter
NG_gyrA	*gyrA*	7	264	121	384	Mutations: S91F, D95A
NG_parC	*parC*	3	332	1	332	Mutation: S87R
NG_23S	23S rRNA gene	100	567	33	599	Wild type

^a^Analysis of *penA* was performed with Ng Star Allele Query (https://ngstar.canada.ca/alleles/query?lang=en)

All other sequence queries were performed with PubMLST “Sequence query—*Neisseria* profile/sequence definitions” using “all loci” (https://pubmlst.org/bigsdb?db=pubmlst_neisseria_seqdef&page=sequenceQuery). The sequences were analyzed together, and the results of this analysis are shown in the table

*NA* no information available

DNA isolated from a pure culture (with the QIAamp DNA Mini Kit from Qiagen, Switzerland) was amplified by primers based on sequences from two internet sites, NG-STAR and PubMLST (see Table 2), and obtained from Microsynth (Switzerland). Sanger sequencing of the purified PCR products was performed at Microsynth (Switzerland) according to their instructions.

## Discussion and conclusions

In this case report, we characterize in detail an unusual multidrug-resistant Ng strain. The demonstrated ceftriaxone-resistant phenotype hinders the recommended dual or monotherapy with ceftriaxone [10].

Penicillin and extended-spectrum cephalosporin resistance has been associated with mutations and recombination within the *penA*, *porinB*, and *ponA* genes and the presence of *bla*; this resistance involves a complex interaction [13].

Interestingly, resistance to penicillin of the analyzed strain was intermediate, although there were two different mutations in the *porinB* gene (cefinase testing was negative). Unfortunately, there is no additional information on allele type 55 at NG-STAR (regarding origin, MICs or epidemiological data). Furthermore, information on the G120N and A121G mutations are available only in combination with other mutations in all other allele types but not as single mutations (based on information from NG-STAR). The combination of G120N and A121G in Switzerland has also not been previously reported in publications with extensive datasets [14].

The *ponA* (penicillin binding-protein 1, PBP1) mutation L421P also seems to be a widespread mutation [5] in Switzerland [14]. According to NG-STAR, this mutation has been observed in Ng with elevated cephalosporin MICs, although the L421P mutation has not been shown to cause resistance in transformation experiments [7]. NG-STAR states the analyzed sequence of allele type 1.

For *penA* (PBP2) allele-type 148 NonMosaic with the A517G mutation, NG-STAR does not provide additional information regarding origin, MICs or epidemiological data. The most recent literature only confirms the dependence of A517G on MICs for extended-spectrum cephalosporins (ceftriaxone and cefixime) [13]. For *penA* (penicillin binding-protein 2, PBP2), diverse sequence variations were observed (i.e., 363 entries for allele types in the NG-STAR database).

The susceptibility to different drugs (including ceftriaxone) may be reduced by the deletion of an adenine in the mtr promoter region [5, 14]; this deletion is also widespread. NG-STAR states the analyzed sequence as allele type 288.

The elevated MIC for the second-line antibiotic ciprofloxacin was due to two different mutations of the *gyrA* gene as well as 1 mutation of the *parC* gene. The combination of *gyrA* (S91F, D95A) and *parC* (S87R) mutations was previously observed in locations other than Switzerland [5, 14, 15]. Two strains with the same combination of *gyrA* and *parC* found in Switzerland [14] had a lower MIC (of 1.5 and 3 mg/l) than the strain in this study (Table 1). This finding makes alternative treatment with fluoroquinolones impossible and makes the strain multidrug resistant.

Azithromycin is below the ECOFF and is consistent with the wild-type allele. This wild-type allele still enables treatment, as suggested by current guidelines [10]. Due to phenotypic susceptibility towards tetracycline, we did not additionally sequence the corresponding genes *tetM* and *rpsJ* [12, 16]. The *bla* gene was not sequenced, but cefinase testing revealed the absence of β-lactamases.

It remains unclear whether this particular strain of Ng was imported from abroad or evolved due to the selective pressure of applied antibiotics or patient noncompliance.

The observed combination of mutations is, to the best of our knowledge, at least very unusual (since it has not been mentioned in recent publications, e.g., [5, 13, 14, 17, 18]) and has not been previously described in Switzerland. Resistance mechanisms remain complex due to the possibility of many combinations of mechanisms [13]. Therefore, our findings contribute to a more extensive knowledge of Ng phenotypes and genotypes, especially since ceftriaxone-resistant strains restrict current treatment guidelines.

## Data Availability

All relevant data are mentioned in the manuscript.
